# A Case of Myxoid Liposarcoma of the Retroperitoneum: A Challenging Tumour for Diagnosis and Treatment

**DOI:** 10.1155/2014/572805

**Published:** 2014-06-12

**Authors:** Emanuele Grasso, Fabio Marino, Michele Bottalico, Michele Simone

**Affiliations:** ^1^Department of General Surgery, Casa di Cura Santa Maria, Via De Ferrariis 22, 70124 Bari, Italy; ^2^Department of Urology, Casa di Cura Santa Maria, Bari, Italy

## Abstract

Retroperitoneal sarcomas are rare neoplasms that account for only 1%-2% of all solid tumors and liposarcomas represent the most frequent histological type. We describe the case of a 44-year-old female with a retroperitoneal myxoid liposarcoma of 22 × 19 × 8 cm in size. The only symptoms were persistent pain and progressive tenderness of the abdomen lasting for two months. The mass was radically excised during laparotomy. CT and MRI were useful to clarify the site of origin of the tumor, relationships with other organs, and planning surgery but final diagnosis was based on histological findings. Here we review the literature about the challenging diagnosis, treatment, and prognostic factors of this disease.

## 1. Introduction

Retroperitoneal sarcomas are rare tumors that account for only 1%-2% of all solid tumors. Most of sarcomas occur outside of the retroperitoneum and only 10%–20% of them originate in the retroperitoneal space. The overall incidence of retroperitoneal sarcomas is estimated at an average of 0.3%-0.4% for 100.000 inhabitants.

They are a group of heterogeneous neoplasms with many histological varieties; the most frequent histological types are liposarcomas (LS), leiomyosarcomas, and malignant fibrous histiocytomas. Liposarcoma is the most common soft tissue sarcoma, accounting for 20% of all sarcomas in adults.

The mortality rates for patients with LS range from 1% to 90%, and recurrence rates range from 5% to 83% depending on the histologic subtype and location [1–3]. Here we report a case of retroperitoneal LS and describe the histological characteristics and the difficulties in the diagnosis.

## 2. Case Report

A 44-year-old female was admitted to our hospital with persistent abdominal pain and a history of progressive tenderness of the abdomen becoming clinically evident during the last two months.

**Figure 1:**
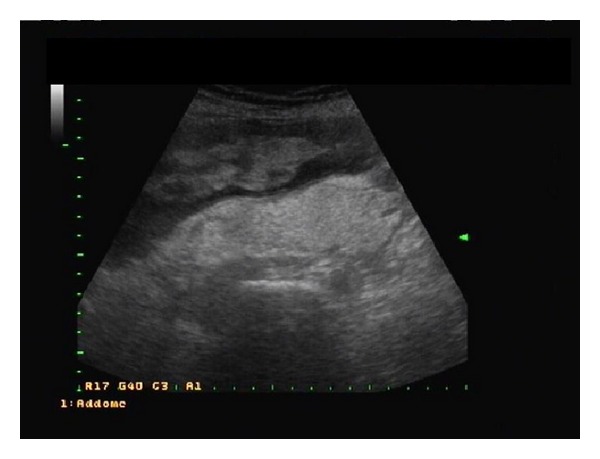
Abdominal ultrasonography showing a solid, heterogeneous retroperitoneal mass with multiple foci of variable size, occupying most of abdominal and pelvic cavity.

Physical examination showed a palpable mass especially extended in the right lower quadrant of the abdomen. Abdominal ultrasonography showed a solid, heterogeneous mass with multiple foci of variable size, probably starting from retroperitoneum, occupying most of abdominal and pelvic cavity and winding the distal 2/3 of right kidney (Figure 1).

Laboratory findings showed a moderate anaemia (haemoglobin level: 102 g/L).

**Figure 2:**
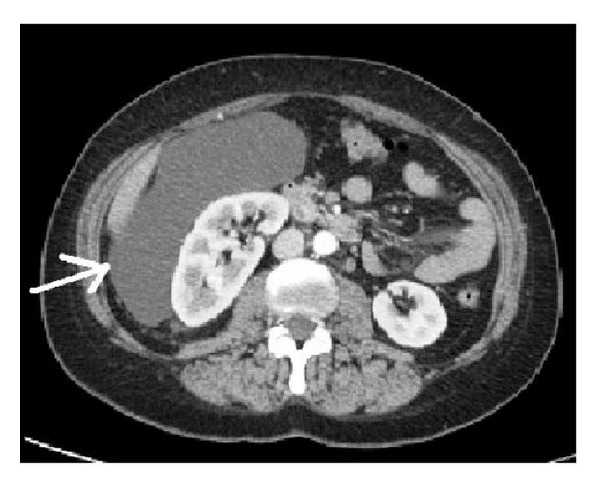
Computed tomography (CT) of the abdomen and the pelvis confirming the presence of a 22 × 19 × 8 cm retroperitoneal mass, extending anteriorly to abdominal wall of right upper quadrant and winding the entire distal right kidney.

Computed tomography (CT) of the abdomen and pelvis confirmed the presence of a 22 × 19 × 8 cm retroperitoneal mass, extending anteriorly to abdominal wall of right upper quadrant and winding the entire distal right kidney without findings of neoplastic infiltration. The tumor displaced the small bowel to the left and the right kidney to the anterior and pushed the uterus to the high quadrant of the abdomen. The mass was well defined from surrounding structures and showed heterogeneous attenuation (Figure 2). Other organs including liver, gallbladder, pancreas, and spleen were within the limit. Abdominal and pelvic magnetic resonance image (MRI) confirmed a retroperitoneal mass close to the right distal kidney.

**Figure 3:**
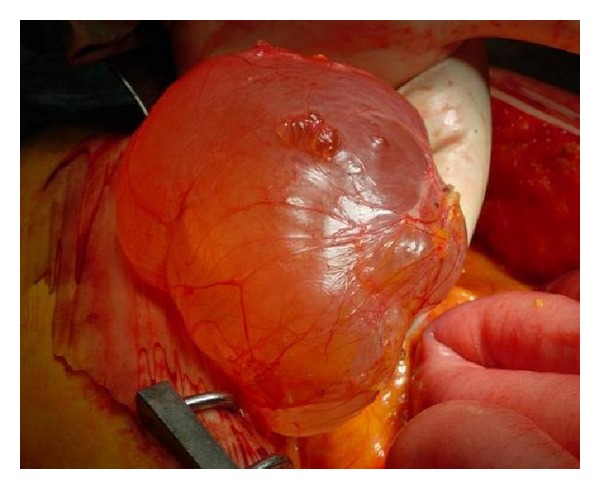
Macroscopic appearance of the mass which is well encapsulated and multilobed. It has gelatinous and rubbery consistency.

At laparotomy, a huge mass was found and completely excised. The tumor showed adhesions to the uterus, urinary bladder, and right kidney, with displacement of the right colon, without infiltration of them. The mass was well encapsulated and multilobed with rubbery and gelatinous consistency and it weighed 1.1 kilograms (Figure 3).

**Figure 4:**
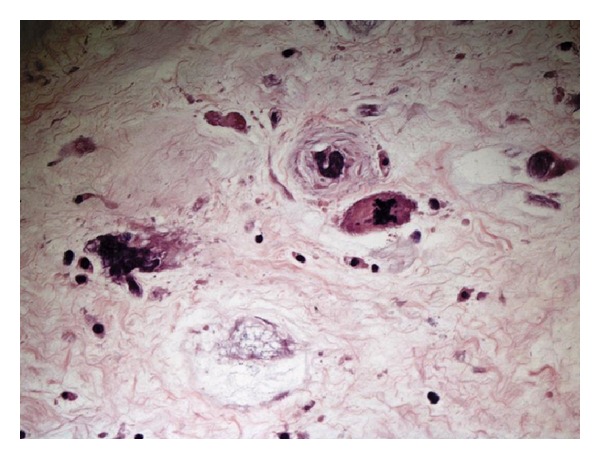
Microscopically, neoplastic mass consists of round to polyhedral cells, which had round, often eccentric nuclei and abundant eosinophilic granular and microvacuolated cytoplasm, myxoid areas, suggesting a histological diagnosis of a retroperitoneal myxoid liposarcoma.

Microscopically, the tumor consisted of round to polyhedral cells, which had round, often eccentric nuclei and abundant eosinophilic granular and microvacuolated cytoplasm, myxoid areas, suggesting a histological diagnosis of a retroperitoneal myxoid LS (Figure 4).

Postoperative course was uneventful and the patient was discharged on the 6th postoperative day.

Radiotherapy and chemotherapy using anthracycline-based therapy were given for 3 months.

After 2 years of clinical follow-up, the patient did not have evidence of disease recurrence.

## 3. Discussion

Retroperitoneal primitive neoplasms are rare accounting for only 0.3%–3% of all tumors. In most cases they are malignant tumors and in 75% of cases they are sarcomas. Liposarcoma is one of the most frequent soft tissue sarcomas found in adults. The main site of origin is the thigh (13–60%) while retroperitoneum is involved in 10–36% of cases [1–3].

The most common histologic subtype is myxoid LS (56.2%), followed by well-differentiated LS (21.9%), including dedifferentiated LS (6.8%), pleomorphic LS (17.8%), and round-cell LS (4.1%) [4].

In the English literature, the majority of patients (93%) had tumors of the well-differentiated/dedifferentiated subtypes. Dedifferentiated LS may be particularly difficult to recognize because they exhibit variable histologic findings but most frequently they resemble unclassified malignant fibrous histiocytoma-like pleomorphic sarcoma or intermediate- to high-grade myxofibrosarcoma [2].

Apart from different histology and malignancy, myxoid/round-cell LS and well-differentiated/dedifferentiated LS are different entities; in more than 95% of myxoid/round-cell LS, a classical t(12; 16) (q13; p11) or t(12; 22) (q13; q12) translocation can be found, which results in fusion of FUS-CHOP or EWSR1-CHOP gene, respectively. Characteristic genetic alteration for well-differentiated/dedifferentiated LS is the amplification of the 12q13–15 region, which includes MDM2 and CDK4 genes [5].

Although APC protein has been shown to be genetically or epigenetically inactivated in a variety of carcinomas, some authors have reported that they have not detected alterations of the APC gene in samples of myxoid/round-cell LS examined. However, they have demonstrated the presence of methylation of the APC promoter that induces a downregulation of the APC transcript [6].

Preoperatively, it is difficult to make definitive diagnosis of retroperitoneal LS because the MRI signal intensity of LS was heterogeneous and varied greatly, depending on the components of the tumour and the different histological patterns. Myxoid LS exhibited low signal intensity on T1W image and high signal intensity on T2W image. Well-differentiated LS presented in high signal intensity on T1W images, intermediate signal intensity on T2W images, and drop-out signal intensity on fat-suppressed MR images; round-cell LS and pleomorphic LS exhibited soft-tissue tumour signal intensity without characteristic of fat signal. Thus, MRI should be an ideal method to diagnose retroperitoneal LS [7].

The retroperitoneal tumors exhibit significant variability in symptoms and pain is the symptom most frequently complained about (60% of cases). Other clinical manifestations may be nausea, vomiting, bleeding, urinary retention, haematuria, dysuria, peripheral paralysis, thrombosis, edema of the lower limbs, weight loss, and fever [1, 4, 8].

Myxoid LS occurs predominantly in the extremities of young adults and has a disproportionately high tendency to metastasize to unusual soft tissue locations, before giving pulmonary metastasis or disseminated spread. Some authors have described cases of myxoid LS in the retropharyngeal space [9], in the right inguinal region presenting as painless inguinal mass [10], in the breast [11], and in the spermatic cord manifesting as painless scrotal or inguinal mass [12] and a primary localization in the pericardium [13]. Other sites of metastasis may be bones, thyroid gland [14], neck, small bowel [15], pericardium, and heart and in very rare cases myxoid LS may metastasize into the pancreas [16].

Jeng et al. report a rare case of retroperitoneal well-differentiated myxoid LS which occurred during pregnancy and was radically excised at the time of cesarean delivery at 36 weeks of gestation [17].

High-grade retroperitoneal sarcomas do significantly worse than low-grade tumors and up to 60% of patients with high-grade retroperitoneal sarcomas die of locally recurrent disease [18, 19]. The majority (78%) of myxoid LS patients developing bone metastases had a histologic high-grade primary tumor [20].

The treatment of myxoid LS needs a team of physicians including surgeons, oncologists, and radiotherapists.

Complete surgical excision is the mainstay of treatment for retroperitoneal sarcomas with emphasis to obtain negative microscopic margins [18–22].

Hatano et al. describe that if a myxoid LS is adjacent to critical structures and wide resection cannot be performed, marginal or intralesional resection combined with postoperative radiotherapy (50–70 Gy, average 59.2 Gy) can achieve a high rate of local control [23]. There are no available results from prospective randomized trials comparing surgery versus surgery plus radiotherapy; however, in a recent review, the authors reported a 5-year local recurrence-free survival after surgery alone and surgery plus radiotherapy in the range of 23–54% and 40–62%, respectively. Furthermore, the respective 5-year rate of overall survival was of 33–49% and 48–64% [20].

The role of chemotherapy has not been well established but its application remains unsatisfactory due to low chemosensitivity of soft tissue sarcomas. Even the first line chemotherapeutic agent doxorubicin only yields a response rate of 18–29%. However, chemotherapy may improve local control and survival [22, 24].

The antibiotic salinomycin, a potassium ionophore, appears to increase the chemosensitivity of sarcoma cell lines to the doxorubicin; therefore, it may be used to decrease the doxorubicin dosage and its toxic side effects [25]. Finally, some authors have published a treatment with Nutlin-3a, an antagonist of MDM2 that stabilized p53 and induced downstream p53 dependent transcription, apoptosis, and growth arrest in LS subtypes cells which overexpress MDM2. Thus, Nutlin represents a promising new therapeutic principle for the treatment of selective groups of sarcomas [26, 27].

The tumor size affects prognoses of patients with intra-abdominal or retroperitoneal liposarcoma; prognoses of patients with large tumors (>20 cm) appear to be significantly poorer than those of patients with small tumors (<20 cm) [28]. Furthermore, vascular involvement seems to be associated with a decreased survival rate [29].

## 4. Conclusion

Our case confirms that retroperitoneal myxoid LS is a challenging tumor for diagnosis and treatment. CT and MRI are essential for assessing the retroperitoneal origin of the tumor, its spread, and planning surgery but final diagnosis was based on histological findings. The overall 5-year survival rate for these neoplasms remains low and surgery appears to be the only option for effective treatment. Chemo- and radiation therapy may improve local control and survival. Tumor size, histologic subtype, grade of tumors, and vascular involvement are important prognostic factors.
